# Impact of high- and low-flow nebulised saline on airway hydration and mucociliary transport

**DOI:** 10.1183/23120541.00724-2022

**Published:** 2023-06-12

**Authors:** Susyn Kelly, Matthew Valentine, Wei Hang Chua, Stanislav Tatkov

**Affiliations:** 1 Fisher & Paykel Healthcare, Auckland, New Zealand; 2 Department of Biomedical Sciences, Ross University, Basseterre, St Kitts and Nevis; 3 School of Health Sciences, Massey University, Palmerston North, New Zealand

## Abstract

**Background:**

Nebulised drugs, including osmotic agents and saline, are increasingly used during noninvasive respiratory support, including nasal high-flow therapy. The authors conducted an *in vitro* study to compare the hydration effect of nebulised isotonic 0.9% and hypertonic 7.0% saline on mucociliary transport.

**Methods:**

In a perfused organ bath, 10 sheep tracheas were exposed to 7.5 mL nebulised 0.9% and 7.0% saline entrained into heated (38°C) and humidified air delivered at high and low flow (20 and 7 L·min^−1^, respectively). Simultaneous measurements of the airway surface liquid height, mucus transport velocity, cilia beat frequency and surface temperature were made over time. The data are presented as mean±sd.

**Results:**

The airway surface liquid height increased significantly with both 0.9% and 7.0% saline: at low-flow by 37.2±10.0 µm and 152.7±10.9 µm, respectively, and at high-flow by 62.3±5.6 µm and 163.4±25.4 µm, respectively (p<0.001). Mucus velocity was increased by both 0.9% and 7.0% saline from a baseline of 8.2±0.8 mm·min^−1^ to 8.8±0.7 mm·min^−1^ and 17.1±0.5 mm·min^−1^, respectively, with low-flow and at high-flow to 9.8±0.02 mm·min^−1^ (p=0.04) and 16.9±0.5 mm·min^−1^ (p<0.05), respectively. Ciliary beating did not change with 0.9% saline, but declined from 13.1±0.6 Hz to 10.2±0.6 Hz and 11.1±0.6 Hz (p<0.05) with 7.0% saline at low- and high-flow, respectively.

**Conclusions:**

The findings demonstrate that nebulised isotonic 0.9% saline, like hypertonic 7.0% saline, significantly stimulates basal mucociliary transport, and the use of high-flow delivery had no significantly different hydration effects compared with low-flow delivery. Hypertonic 7.0% saline suppressed ciliary beating, indicating an increase in airway surface liquid osmolarity, which may have negative effects on the airway surface with frequent use.

## Abstract 2

**Nebulised isotonic saline, delivered with high flow of humidified air in a perfused tracheal model, hydrates the airway surface and increases the mucus transport velocity without suppressing beating cilia as observed with hypertonic saline** https://bit.ly/3KU5HOc

## Introduction

Large conducting airways are lined by a continuous ciliated epithelium with an overlying airway surface liquid (ASL) consisting of periciliary and mucus layers that protect the airway from desiccation and infection. The periciliary layer is composed of a less viscous fluid, which facilitates beating of motile cilia. Overlaying the periciliary layer is a mucous blanket that contains more viscous mucins secreted from specialised respiratory epithelial cells and glands. The mucus traps inhaled foreign particles and micro-organisms, which are moved in the mucus by beating cilia to the larynx for swallowing. To prevent evaporation from the ASL and maintain a thermodynamic balance, inspired air is heated to body temperature and humidified to pressure saturation (BTPS) in the upper airway, which is known to be essential for mucociliary transport [1].

In muco-obstructive airway diseases, mucins are hypersecreted into the ASL [2]. This results in an insufficiently hydrated ASL with compromised physiological viscoelastic properties, which slow mucociliary transport and lead to mucostasis and cough [3]. Nebulised liquid formulations are increasingly being used in noninvasive respiratory support such as nasal high-flow therapy because of the simplicity of application and tolerance by patients related to humidification of the delivered gas [4]. However, aerosols entrained in a high-flow system of humidified air can be susceptible to condensation growth [5, 6] and can become diluted by high flow and are thus less effective.

Various osmotic agents are used to decrease the viscosity of accumulated airway mucus [3]. Radioactive aerosols have been shown to be cleared more rapidly in cystic fibrosis patients following administration of nebulised hypertonic saline (HS) solutions in a concentration-dependent fashion [7]. However, increasing HS concentration frequently leads to patients reporting pharyngeal irritation [7], likely to result in a lower tolerance of this therapy. Inhalation of nebulised isotonic saline (IS) improves clinical signs and lung function in children with cystic fibrosis [8] and decreases breathlessness scores in patients with COPD [9].

Little is known about how nebulised IS and HS solutions are effective, the untested rationale being that the osmotic agents act through “moistening of the airway surface” [9], hydrating the ASL [10] and therefore resulting in improved mucociliary transport [7]. A recent multicentre trial in children with cystic fibrosis aged 3–6 years showed that the use of nebulised HS twice daily for 1 year had a positive effect on structural lung changes [11]. A double-blind, randomised controlled crossover trial in patients with primary ciliary dyskinesia found no significant improvement in quality of life after twice-daily inhalations of 7% HS or IS for 12 weeks [12]. However, adverse events were more frequent with HS. The authors believe the lack of improvement in quality of life could be due to the control treatment with IS also having therapeutic effects on cilia and ASL [13]. The authors also note that the oral administration of saline may not be the best method of delivery, as upper airways are also commonly affected in this heterogeneous disease. *In vitro* studies have shown that nebulised HS increases the ASL [10] and suggest that the deposition of HS causes osmotic changes that lead to an increase in transepithelial water flow through respiratory epithelial cells which act as osmometers for the regulation of ASL [14]. Administration of HS has been shown to result in a rapid (<5 min) reduction of the epithelial cell heights which persists for >4 h [10]. The ciliary beat frequency (CBF) has been reported to decrease immediately after exposure to HS, but not IS or hypotonic saline, and results in cilia stasis within 5 min [15]. This has been associated with histological changes in the epithelium, indicative of cell damage from fluid transport towards the surrounding medium. It is not known how those findings are applicable to nebulised saline delivered with high flow.

The study was designed to test a hypothesis that both nebulised IS and HS solutions of sodium chloride (NaCl) hydrate the ASL and stimulate mucociliary transport when delivered with high flows of humidified air.

## Methods

**FIGURE 1:**
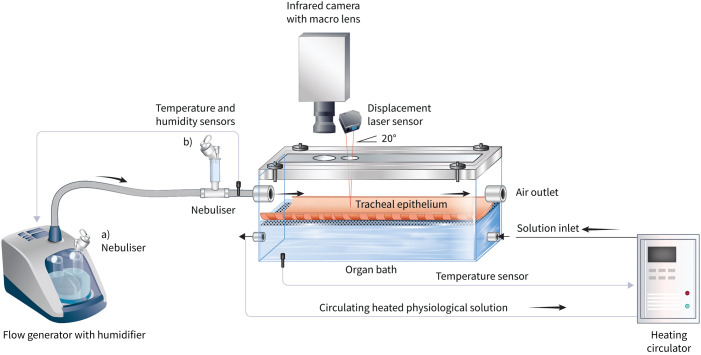
Experimental set-up to study the effects of nebulised isotonic (IS) (0.9% sodium chloride (NaCl)) and hypertonic (HS) (7.0% NaCl) saline delivered in air at body temperature and pressure saturated condition (38°C, dew point 38°C) at low (7 L·min^−1^) and high (20 L·min^−1^) flow rates over the airway surface. The sheep trachea was mounted flat in an organ bath with the epithelium facing upwards. The IS and HS solutions were nebulised with a vibrating mesh nebuliser (7.5 mL over a 15-min period, in accordance with clinical practice) at a) the humidification chamber outlet port with high flow to mimic delivery during noninvasive respiratory support and b) the organ bath with low flow. A high-speed infrared camera with a macro lens was used to measure the airway surface temperature, mucus transport velocity and ciliary beat frequency on the airway surface. A laser displacement sensor was used to measure changes in the airway surface liquid height for the duration of the experiments.

Full materials and methods are presented in the supplementary material. 10 tracheas, harvested from healthy sheep immediately after slaughter, were placed in the experimental set-up (figure 1) to measure changes on the airway surface in response to nebulised saline solutions, entrained into BTPS air at 7 L·min^−1^ and 20 L·min^−1^, mimicking low- and high-flow delivery systems, respectively. Baseline measurements were made over the first 15 min with just BTPS air. As reported previously [1], mucociliary transport, in terms of mucus transport velocity (MTV) and CBF, on the airway surface was measured with infrared macroimaging, which allowed the authors to measure the airway surface temperature for monitoring thermodynamic changes. ASL height was simultaneously and continuously monitored using a laser displacement sensor. The nebulised saline and the effect of relative humidity on the particles’ size during high-flow delivery were characterised by measuring the mass median aerosol diameter (MMAD) with an optical particle sizer, when the nebuliser was connected to the humidification chamber outlet (figure 1a). The MMAD is defined as the diameter at which 50% of the particles of an aerosol by mass are larger and 50% are smaller. Aerosol delivery with a flow of 7 L·min^−1^ was not measured, due to the technical limitation of the method given the high concentration of particles in the optical path and oversaturation of the signal.

Two tracheas were used for morphology to measure the potential effect of IS and HS on the tracheal mucosal height over time. The airway surface was directly exposed to IS and HS solutions, then a 4-µm section of each was placed in 10% formalin after 0, 5, 10 and 15 min, 24 h prior to routine histological processing and preparation with haematoxylin and eosin stain for examination under a light microscope. The heights of three regions of intact mucosae (minimal artefact) on each tissue section were measured.

GraphPad Prism (V8.3.0) was used for statistical analysis of measurements. Significance testing was performed with a two-tailed paired t-test as appropriate, and a two-way ANOVA Tukey test where p<0.05 was statistically significant. All data were tested for normality with the D'Agostino–Pearson test and presented as mean±sd.

## Results

**FIGURE 2:**
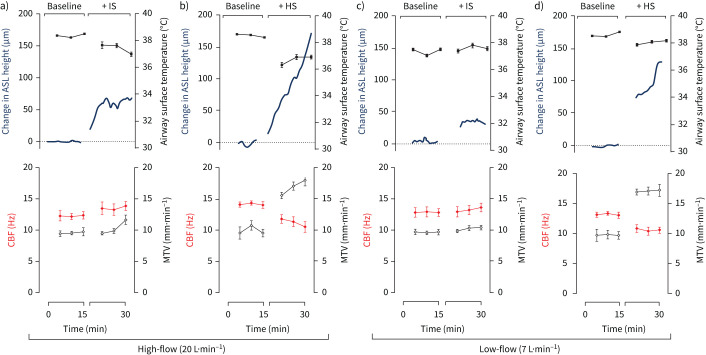
Representative real-time changes in airway surface parameters with nebulised saline solutions delivered onto sheep tracheas at high-flow (20 L·min^−1^) and low-flow (7 L·min^−1^). Measurements of the airway surface liquid (ASL), mean airway surface temperature, ciliary beat frequency (CBF) and mucus transport velocity (MTV) were made with a high-speed infrared macroimaging camera and laser displacement sensor during a 15-min period. Baseline data for each parameter were collected over the first 15-min recording period when the mucosal surfaces of the tracheas were exposed to body temperature and pressure saturated (BTPS) air (38°C, dew point 38°C). Over the next 15-min recording period, parameters were measured when the tracheas were exposed to nebulised isotonic saline (IS) (0.9% sodium chloride (NaCl)) at a) high-flow and c) low-flow or hypertonic saline (HS) (7.0% NaCl) nebulised at b) high-flow or d) low-flow.

**FIGURE 3:**
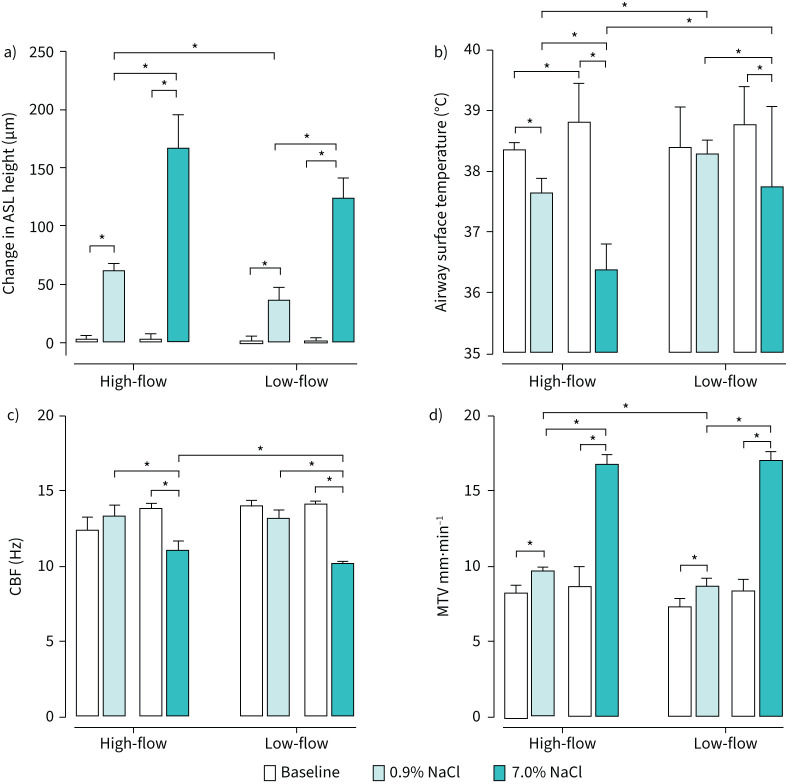
Mean changes in airway surface parameters with nebulised saline solutions delivered onto sheep tracheas at different flow rates. Surface parameters measured were a) airway surface liquid (ASL) height, b) airway surface temperature, c) ciliary beat frequency (CBF) and d) mucus transport velocity (MTV). Mean baseline values for each parameter were recorded with air at body temperature and pressure saturated (38°C, dew point 38°C) at high- (20 L·min^−1^) and low-flow (7 L·min^−1^) particle delivery rates. Values with nebulised isotonic (0.9% sodium chloride (NaCl)) and hypertonic (7.0% NaCl) saline solutions were also measured at low and high flow rates. *: p<0.05.

Representative real-time changes in the airway surface parameters are presented in figure 2. Mean changes of the airway surface parameters are presented in figure 3.

### Baseline

During baseline data collection, when the airway surface was exposed to BTPS air at high-flow (20 L·min^−1^), the average ASL height (3.3±3.3 µm), airway surface temperature (38.6±0.4°C), MTV (8.5±0.9 mm·min^−1^) and CBF (13.2±0.5 Hz) were within normal ranges described previously [16] and were not significantly different from the baseline measures at low-flow (7 L·min^−1^) (figure 3) (supplementary material, part 2).

### High-flow with IS

When entraining IS into high-flow BTPS air, the average ASL height and MTV increased significantly, 20-fold to 62.3±5.7 µm (p<0.001) and by 15% to 9.8±0.2 mm·min^−1^ (p<0.05), respectively. In contrast, the airway surface temperature declined significantly to 37.7±0.2°C (p<0.05) (figure 3b). CBF did not change significantly. Real-time data from a representative experiment (figure 2a) showed that the ASL height increased rapidly in the first 5 min of IS nebulisation before stabilising.

### High-flow with HS

Exposing the airway surface to HS entrained into high-flow BTPS air resulted in a significant increase (p<0.001) from baseline in the average ASL height (167.2±29.0 µm; a 55-fold increase) and MTV (16.9±0.5 mm·min^−1^; a two-fold increase) (figure 3). There was a significant decline (p<0.05) in the average airway surface temperature (to 36.4±0.4°C) and CBF (to 11.1±0.6 Hz) compared to baseline values. The real-time data from a representative experiment (figure 2b) demonstrated that the ASL height increased continually over the recording period.

### Low-flow with IS

The introduction of nebulised IS into low-flow BTPS air resulted in a significant increase in the average ASL height (to 37.2±10.0 µm; p<0.001) and average MTV (by 10% to 8.8±0.4 mm·min^−1^; p<0.05). There were no significant changes to the average airway surface temperature or average CBF following IS administration (figure 3).

### Low-flow with HS

Entraining HS into low-flow BTPS air resulted in similar findings to the high-flow results. There was a significant increase (p<0.001) in the average ASL height (by 40-fold to 124.7±16.6 µm) and average MTV (two-fold to 17.1±0.5 mm·min^−1^) with a significant decline in airway surface temperature (to 37.8±1.3°C; p<0.05) and CBF (to 10.2±0.1 Hz; p<0.05) (figure 3).

### Nebulised saline particle size

**FIGURE 4:**
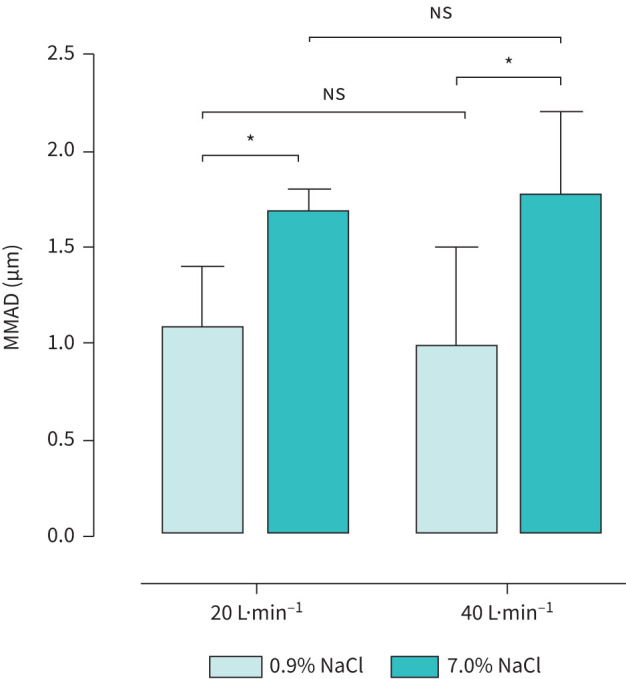
Change in mass median aerosol diameter (MMAD) of nebulised isotonic (0.9% sodium chloride (NaCl)) and hypertonic (7.0% NaCl) solutions when delivered with body temperature and pressure saturated carrier air at high flow rates (20 and 40 L·min^−1^) *via* a heated breathing circuit. ns: nonsignificant. *: p<0.05.

MMADs of the nebulised IS and HS solutions are presented in figure 4 and in the supplementary material (part 3). At high-flow (20 L·min^−1^) the concentration of the nebulised saline had a significant effect on particle size, with the MMAD of IS particles being smaller (1.1±0.3 µm) than those of HS particles (1.7±0.1 µm) when delivered with BTPS air. Increasing the flow rate from 20 to 40 L·min^−1^ did not have a significant effect on the IS or the HS particle size. However, changing the carrier air temperature and humidity had a significant effect on particle sizes, except when nebulised IS was exposed to air with 80% and 60% relative humidity (supplementary material; part 3).

### Histology

**FIGURE 5:**
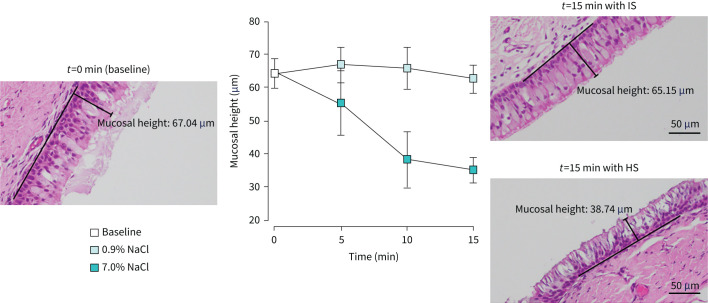
Representative histological images showing the mean (n=6) mucosal height (labelled in images) of tracheal epithelial cells at baseline (*t*=0) and after *t*=5, 10 and 15 min of exposure to isotonic (0.9% sodium chloride (NaCl)) and hypertonic (7.0% NaCl) saline solutions. The chart demonstrates a reduction of mucosal height caused by the hypertonic saline solution. Scale bars=50 µm.

There was a significant (p<0.05) decrease in the mucosal height of the tracheas exposed to liquid HS from 64.4±4.5 µm to 35.2±3.7 µm after 15 min; a 45% decline (figure 5), whereas there was no change in mucosal height for the tracheas immersed in IS after 15 min.

## Discussion

**FIGURE 6:**
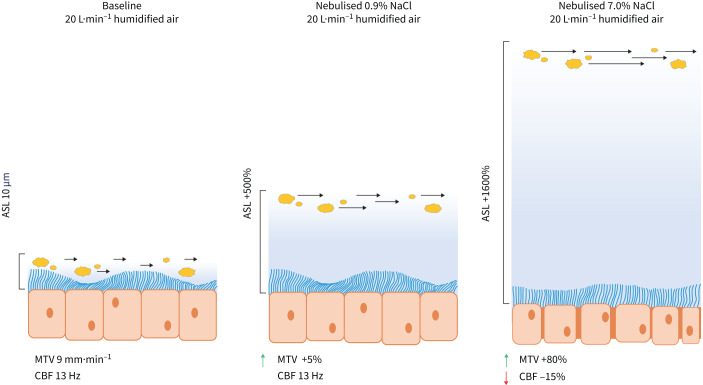
A summary of the key findings. Nebulised isotonic 0.9% saline delivered with 20 L·min^−1^ of humidified air increased the airway surface liquid (ASL) by ∼500%, assuming the basal ASL thickness at baseline is 10 µm, and accelerated mucus transport velocity (MTV) by 5% without any effect on ciliary beat frequency (CBF). Hypertonic 7.0% saline increased the ASL by ∼1600% and MTV by 80%, but caused a reduction of CBF by 15%, which may indicate a ciliostatic effect.

The results demonstrate that nebulised IS and HS significantly increased ASL height, stimulating mucociliary transport by increasing MTV, and the high-flow delivery of nebulised saline did not reduce this hydration effect. HS, relative to IS, increased ASL height three-fold (167.2±29.0 µm *versus* 62.3±5.7 µm when delivered at high flow and to 124.7±16.6 µm *versus* 37.2±10.0 µm when delivered at low-flow) and doubled MTV, but suppressed CBF. A summary of the key findings is presented schematically in figure 6. The transitory ciliostatic effect from HS exposure could be due to ASL hyperosmolarity, removing water from the epithelial cells causing dysfunction. Contrary to HS, the nebulised IS can be considered optimal for hydration of the airway surface without suppressing ciliary function.

That the basal mucociliary transport parameters, measured as MTV and CBF, were stable and comparable to previous reports [1, 16–18], indicate the thermodynamic balance during exposure to flowing heated and humidified air to BTPS over the epithelium. There was a small but significant decline in airway surface temperature when nebulised saline was introduced in spite of the precise control of BTPS condition of airflow that carried the aerosol. The temperature drop probably occurred as a result of water condensation on the ASL surface, according to Nusselt's film theory [19]. To determine the thermal effect, the heat transfer across the ASL was estimated (supplementary material, part 4) and suggested that the underlying epithelium was insulated from the decreased surface temperatures by the large increases in the ASL height associated with the administration of the nebulised saline. The protecting effect of ASL on the tracheal epithelium is confirmed by a poor correlation between the airway surface temperature and the MTV (R^2^=0.15) and CBF (R^2^=0.10); this suggests that there were no inhibitory effects on the basal mucociliary transport, which is known to be sensitive to changes in temperature [20].

Nebulised IS had an immediate effect on the ASL height, increasing it 20-fold, consistent with a previous report that the ASL increased by 20 µm when human bronchial cells cultures were exposed to nebulised IS for 15 min [10]. The increases in ASL following administration of saline might be due to deposition of the NaCl particles and associated water, and/or as a result of osmotic activity drawing fluid from the underlying epithelial cells. An approximation of the ASL height changes during nebulisation of IS (supplementary material, part 3) revealed that the measured ASL height could be achieved from just the nebulised IS particles depositing on the surface, with the ASL remaining iso-osmolar during the nebulisation, and without triggering any large transepithelial water fluxes.

The changes in the ASL height were strongly correlated with MTV (R^2^=0.93), which supports a role of hydration in mucociliary transport. Similar to a previous report [10], the administration of HS had an immediate effect on the ASL height; however, the increase was considerably greater than that seen with IS. Hyperosmolarity of the ASL has been shown to trigger the release of mediators and histamine [21] and mucin hypersecretion [22]. Mucin secretion and water absorption is a very rapid process; the individual mucin granules release their contents in only 0.1 s [23] and these absorb several hundred-fold their mass in water, resulting in an increase in mucus volume within a second [24]. Although increases in the ASL have been associated with increased rates of mucociliary transport in the upper airways [25], such changes might be harmful in the smaller airways causing obstruction [24]. During exposure to HS, the ASL height did not stabilise in the measurement period (figure 2b and d) and indicates that the ASL height may keep rising if nebulisation continued, which could lead to excessive fluid in the airways, occlusion of small bronchi, and cough. This would seem to be especially the case in patients with muco-obstructive disease [2] with mucin hypersecretion, which has been linked to increased ASL osmotic pressures and reduced mucociliary transport [26].

CBF did not change with IS administration, but it was decreased significantly with exposure of the epithelium to nebulised HS at both low- and high-flow rates. The decrease in the CBF with HS was associated with an increase in MTV. Although CBF has been reported to be directly related to MTV under physiological conditions [27], this relationship is not present in HS [1, 16] and during an exposure of the airway surface to unidirectional flow of cooler and drier air (supplementary material, part 5). This disproportional relationship was explained by the shear thinning properties of mucus [1], where the sliding of the mucus layer during both effective and recovery strokes of the beating cilia allowed the MTV to far exceed the displacement cilia could impart [1, 16]. In the current study, a substantial increase of ASL height by the nebulised HS was caused by an increased osmolarity, which is known to decrease CBF [28]. The hyperosmolarity could result in excessive fluid transport out of the cells, as reported previously [10, 29, 30], and demonstrated by the 43% decrease in mucosal height that was found following direct exposure of the tracheal epithelium to a HS solution (figure 5). Damage to the epithelial cells [15], or dysfunction resulting from the cell shrinkage, might have contributed to the decreased CBF. Such changes to the epithelial cell layer might also explain the pharyngeal irritation side-effect reported with HS nebulisation [7].

Cilia movement is powered by dynein, a cytoskeletal motor protein that uses ATP hydrolysis for energy. This generates force and movement on microtubules, leading to cilia bending [31]. The dynein ATPase activity of the axoneme regulates CBF, and a reduction in CBF has been well established [32]. In a study on the effects of osmotic agents, Nadesalingam *et al*. [33] found that HS suppresses neutrophil extracellular trap formation and promotes apoptosis by reducing nicotinamide adenine dinucleotide phosphate oxidase activity. The authors suggest that neutrophil dehydration, caused by equimolar concentrations of choline chloride, a-mannitol and d-sorbitol, produced the same suppressive effect. However, a recent study by Mazzitelli *et al*. [34] found the opposite, concluding that high salt concentration, rather than osmolarity, led to activation of the neutrophil response. Regardless of the mechanism, it can be speculated that ATP hydrolysis in ciliated epithelial cells may also be affected by HS and reduce CBF.

Smaller MMAD of IS relative to HS particles (figure 4), which did not change when flow was doubled from 20 to 40 L·min^−1^, may also indicate an advantage of delivery of the nebulised IS to distal airways during noninvasive respiratory support. Reduction of relative humidity of air in the breathing circuit may reduce MMAD of the nebulised IS (supplementary figure S1), which may further increase the efficiency of high-flow delivery of saline. Additionally, control of relative humidity in the high-flow system can potentially be used for targeted delivery, as smaller particles tend to deposit more distally in the bronchial tree [31, 35].

The study has several limitations. The excised trachea from healthy sheep may not truly represent the pathology in humans with mucus hypersecretion. However, the whole trachea maintained in the organ bath and ventilated with air conditioned to BTPS allowed the thermodynamic balance to be maintained on the mucosal surface, thereby excluding any effects of evaporation on the mucosal surface that may occur during inhalation of unconditioned air. While the results demonstrate that nebulised IS significantly increases the ASL and MTV in the basal mucociliary transport, the effects still need to be confirmed in studies on patients with muco-obstructive airways disease. Nevertheless, it appears unlikely that the negative effect of HS on the ciliated cells could not be present in patients with muco-obstructive diseases based on the low tolerance reported in the literature [7]. The unidirectional flow did not truly represent the tidal breathing, when heat and moisture are recovered during expiration, and kinetics on the nebulised particles would greatly depend on the breathing pattern. The use of unidirectional flow meant that relatively low-flow settings were used in the experimental protocol, which can be viewed as a major limitation. During normal tidal breathing of unconditioned room air *via* a mouth T-piece, tracheal or nasal cannula interface, flow is expected to exceed 20 L·min^−1^ in the trachea of an adult; however, no significant thermodynamic changes are observed on the ASL because of heat and moisture recovery following expiration [1]. To incorporate tidal breathing in this *in vitro* experimental set-up, it would require a complex lung simulator with heated and humidified air involving the complex control. The airway epithelium in the perfused organ bath is very sensitive to reduced temperature and humidity of airflow, as demonstrated previously in a similar experimental model [14]. Flows of 7 L·min^−1^ and 20 L·min^−1^ allowed the authors to investigate the effect of a three-fold increased dilution of nebulised IS and HS delivered to the tracheal epithelium. Nebulisation of saline at flows >20 L·min^−1^ and the effect of repeated nebulisations have not been tested in the model, but these research questions could be addressed in future clinical studies. Deposition of the nebulised saline particles on a flat tracheal surface could be different from the deposition on the surface of the circular-shaped organ, but the set-up allowed several key physiological parameters to be measured, including real-time changes in ASL height. The histological examination was performed on tissue exposed to liquid forms of IS and HS to demonstrate the maximum effect of HS on the epithelial cells over a typical nebulisation period, allowing for the maximum increase of the ASL to be estimated. According to the study protocol, the measurements were performed only during the nebulisation period (15 min) and reabsorption of the increased ASL volume after nebulising was not studied. There are reports of the ASL returning to initial levels within 60 min [10]. Finally, the effect of relative humidity of the carrier air transporting the nebulised saline on mucociliary transport and ASL was not studied, but a reduction of humidity and the particle size changes through condensation growth [36, 37] was investigated. This may help to target deposition in the airway, particularly during delivery of the nebulised solutions with nasal high flow where larger particles are retained in the nasal passages [5] (supplementary material, part 3). The analysis showed that the carrier air condition had a significant effect on the particle size, while the airflow rate did not.

The study demonstrates that the high-flow *versus* low-flow delivery did not reduce the hydration effect of the nebulised saline, and IS, like HS, significantly increases the ASL height and MTV. Although HS appeared to produce a greater increase of MTV and ASL height, these changes might have been associated with epithelial cell dysfunction and may inflict damage in the long term. The study may serve as a rationale for further investigations into the frequent use of nebulised IS or other physiological solutions that could be delivered with nasal high-flow or any alternative forms of noninvasive respiratory support in patients with muco-obstructive diseases and delayed mucus clearance; particularly when the upper airways are affected, which is common in cystic fibrosis and primary ciliary dyskinesia.

## Supplementary material

10.1183/23120541.00724-2022.Supp1 **Please note:** supplementary material is not edited by the Editorial Office, and is uploaded as it has been supplied by the author.
Supplementary material 00724-2022.SUPPLEMENT
