# Supplementary material for: Impact of high- and low-flow nebulised saline on airway hydration and mucociliary transport
Source: ERJ Open Res. 2023 Jun 12;9(3):00724-2022. doi: 10.1183/23120541.00724-2022 (PMC10258716; doi:10.1183/23120541.00724-2022)
Supplement: Supplementary file 1 [file 00724-2022.SUPPLEMENT.pdf]

# Impact of high- and low-flow nebulized saline on airway hydration and mucociliary transport

*S Kelly*[1], *M Valentine*[2], *W Chua*[3] *and S Tatkov*[1]*

[1] Fisher & Paykel Healthcare, Auckland, New Zealand.

[2] Department of Anatomy and Pathology, Ross University, St Kitts, West Indies.

[3] School of Health Sciences, Massey University, Palmerston North, New Zealand.

* Corresponding Author Stanislav.Tatkov@gmail.com

# SUPPLEMENTAL MATERIAL

Part 1: Detailed materials and methods

Part 2: Summary of results

Part 3: Particle condensation growth measurements and mathematical modelling to determine volume changes from osmolarity and particle deposition on the tracheal surface with nebulized saline solutions

Part 4: Mathematical modelling to estimate the thermal effects from condensation on the surface and changes to the airway surface liquid (ASL) film height

Part 5: Effect of cooler and drier air on the ASL and mucociliary transport

## Part 1: Detailed materials and methods

### Samples

Ten tracheas, harvested from apparently healthy sheep immediately after slaughter, were collected from an abattoir and transported at room temperature to the laboratory. The tracheas were opened along the ventral mid-line and fixed flat with the epithelium positioned upward in a previously described[1] heated (38 °C) organ bath (Figure 1). Time from collection at the abattoir to the beginning of the laboratory experiments was around 30 minutes for each trachea.

### Equipment

A high-flow generator with humidifier (Airvo™ 2, Fisher & Paykel Healthcare, New Zealand) was used to create a unidirectional flow of heated and humidified air over the epithelial surface of the tracheas at 7 L/min (low-flow delivery) to mimic inhaler delivery or 20 L/min (high-flow delivery) to mimic nasal high-flow delivery. The temperature and dew point of the air were controlled using external sensors at the inlet of the organ bath (HMP110, Vaisala, Finland). To establish the effects of nebulized saline solutions, either IS (7.5 mL of 0.9% NaCl) or HS (7.5 mL of 7.0% NaCl) was nebulized into the air path using a vibrating mesh nebulizer (Solo, Aerogen, Ireland) over a 15-minute period, according to clinical practice. The nebulizer was connected to the humidification chamber (Figure 1A) throughout high-flow delivery to mimic nebulization during high-flow therapy via tracheostomy or nasal cannula. The nebulizer was connected directly to the organ bath (Figure 1B) to mimic low-flow delivery as would occur with a typical handheld nebulizer device.

The top cover of the organ baths contained heated calcium fluoride ($CaF_2$) optical windows, one of which was used to continuously measure the ASL with a displacement laser sensor (LK-G32 and LK-Navigator software, Keyence Corporation, Japan). This sensor is based on the light-scattering principle and has repeatability of up to 0.05 µm. Its analogue

signal was recorded using Keyence software (LK-Navigator, Japan). The other optical window was used for simultaneous infrared radiation (IR) high-speed macro-video imaging (SC7000 camera and L0905 lens, FLIR, US) to measure MTV, CBF and surface temperature, as reported in detail previously[2]. The IR macro-video imaging was carried out for 30 seconds, every 5 minutes, during the 30-minute experiment, at 100 frames/s with a field of view of 9.6 x 7.7 mm.

Baseline measurements were made over the first 15 minutes with air heated to 38 °C and fully saturated with water, so-called "body temperature pressure saturated" (BTPS), on all tracheas (N = 10). Thereafter measurements were made at 15-minute intervals with exposure to IS (N = 5) and HS (N = 5).

GraphPad Prism (V8.3.0, US) was used for statistical analysis of measurements. Significance testing was performed using a two-tailed paired t-test, and two-way ANOVA Tukey test where $p < 0.05$ was statistically significant. All data was tested for normality with the D'Agostino & Pearson test.

The mass median aerosol diameter (MMAD) was measured with an optical particle sizer (Model 3330, TSI, Germany) at flows of 20 and 40 L/min with the nebulizer connected to the humidification chamber outlet (Figure 1A), using a restrictor to prevent the optical receiver from becoming over-saturated. This enabled investigation into the effect of relative humidity on the size of particles during high-flow treatment. Nebulized saline was entrained into the following carrier air conditions to see the effect of air conditions on MMAD: temperatures (37 °C, 41 °C and 47 °C) and dew points (28 °C, 33 °C and 37 °C) corresponding to relative humidity (60%, 80% and 100%). Low-flow particle delivery (7 L/min) generated particles in too great a concentration for the optical receiver in the particle sizer to provide meaningful measurements and therefore was not tested. The particle sizer sampled gas at the end of the standard nasal cannula interface (OPT944, Fisher &

Paykel Healthcare, New Zealand) used in clinical practice during nasal high-flow (NHF) therapy (Airvo 2, Fisher & Paykel Healthcare, New Zealand). The particle count was based on particle sizes of between 0.3 and 10 µm, where the MMAD is the spherical aerodynamic equivalent diameter with the same physical properties at which half the mass lies below the stated diameter.

For histology, tracheas were collected within 20 minutes of killing at the local abattoir, the “plucks” from four freshly-killed adult Barbados black-belly sheep were removed and N = 2 tracheas were submerged in 1 L of IS (0.9% NaCl) solution and N = 2 in 1 L of HS (7.0% NaCl) solution. The tracheal segments were sharply excised into 4 µm sections and placed in 10% formalin after 0, 5, 10 and 15 minutes for fixation for a minimum of 24 hours prior to routine histological processing and preparation with haematoxylin and eosin stain. Sections were examined using a light microscope (Olympus BX51, Japan) at 200x and the heights of three regions of intact mucosae (minimal artefact) of each tissue section were measured using an Olympus DP27 camera and cellSens imaging software to determine a mean height.

## Part 2: Summary of results

The table below summarizes all the experimental air conditions and nebulized saline delivery and the resulting mucociliary transport, surface temperatures and airway surface liquid levels.

**Supplemental Table 1:** Mean airway surface measurements and particle size distributions measured over a 15-minute period while the sheep tracheas were exposed to different nebulized saline solutions: airway surface liquid (ASL), mucus transport velocity (MTV), cilia beat frequency (CBF) and mass median aerosol diameter (MMAD). ^ dew point temperature measurements could not be made due to the proximity of the nebulizer to the dew point temperature sensor. ~ low-flow nebulization generated particles in too great a concentration for the optical receiver in the particle sizer to provide meaningful measurements and therefore was not tested. * values significantly different to measurements made under body temperature and pressure saturated (BTPS) conditions ($p < 0.001$).

| **Parameters** | **High-flow Nebulizing (20 L/min)** | | | **Low-flow Nebulizing (7 L/min)** | | |
| --- | --- | --- | --- | --- | --- | --- |
| | ***BTPS*** | **Isotonic** | **Hypertonic** | ***BTPS*** | **Isotonic** | **Hypertonic** |
| Air temperature (°C) | *38.1 (0.6)* | 38.2 (0.1) | *38.1 (0.6)* | *38.1 (0.6)* | 38.0 (1.9) | 38.1 (0.2) |
| Dew point (°C) | *37.3 (0.5)* | 37.2 (0.5) | *37.3 (0.5)* | *37.3 (0.5)* | ^ | ^ |
| Airway surface temperature (°C) | *38.6 (0.4)* | 37.7(0.2) | *38.6 (0.5)* | 38.6 (0.2) | 38.3(0.2) | 37.8 (1.3) |
| ASL height (µm) | *3.3 (3.3)* | 62.3 (5.6)* | *167.2 (29.0)* | 0.8 (3.7) | 37.2 (10.0)* | 124.7 (16.6) |
| MTV (mm/min) | *8.5 (0.9)* | 9.8 (0.2)* | *9.4 (2.0)* | 7.9 (0.6) | 9.8 (0.7)* | 17.1 (0.5)* |
| CBF (Hz) | *13.2 (0.6)* | 13.4 (0.6) | *13.1 (0.6)* | 14.2 (0.2) | 13.3 (0.5) | 10.2 (0.1)* |
| MMAD (37 °C, dew point 28 °C) | - | 0.4 (0.1) | - | - | - | - |
| MMAD | - | 0.5 (0.1) | - | - | - | - |

| | | | | | | |
|---|---|---|---|---|---|---|
| (37 °C, dew point 33 °C) | | | | | | |
| MMAD<br>(37 °C, dew point 37 °C) | - | 1.1 (0.3) | - | - | - | - |
| MMAD<br>(41 °C, dew point 37 °C) | - | 1.0 (0.4) | - | - | - | - |
| MMAD<br>(47 °C, dew point 37 °C) | - | 0.8 (0.4) | - | - | - | - |

## Part 3: Particle condensation growth measurements and mathematical modelling to determine osmolarity changes on the tracheal surface with nebulized saline solutions

Particle condensation growth[3] appears to be an important factor in determining the deposition rates of nebulized saline solutions onto different parts of the airways. Particles larger than 5 µm in aerodynamic diameter tend to deposit in the upper airway while smaller particles (< 2 µm) are more likely to deposit in the lower airway[4]. Nebulized NaCl reduces the water vapour pressure on the particle surface, resulting in condensation growth at relative humidities at or below saturation conditions[5]. Condensation growth offers an opportunity for adjusting particle concentration and sizes, allowing for target deposition in the airways[6]. In this study particle sizes were measured from nebulized isotonic and hypertonic saline solutions with different carrier air conditions and flow rates to determine what effect the carrier air conditions have on particle size and condensation growth.

### Method

Measurement of particle sizes at temperatures (37 °C, 41 °C and 47 °C) and dew points (28 °C, 33 °C and 37 °C) corresponding to relative humidity (60%, 80% and 100%) was performed with an optical particle sizer (Model 3330, TSI, Germany) at flow rates of 20 and 40 L/min with the nebulizer connected to the humidification chamber outlet (Figure 1A), using a restrictor to prevent the optical receiver from becoming over-saturated; this enabled

investigation into the effect of relative humidity on the size of particles during high-flow treatment. Low-flow particle delivery (7 L/min) generated particles in too great a concentration for the optical receiver in the particle sizer to provide meaningful measurements and therefore was not tested. The particle sizer sampled gas at the end of the standard nasal cannula interface (OPT944, Fisher & Paykel Healthcare, New Zealand) used in clinical practice during nasal high-flow (NHF) therapy (Airvo 2, Fisher & Paykel Healthcare, New Zealand). The particle count was based on particle sizes of between 0.3 and 10 µm, where the mass median aerodynamic diameter (MMAD) is the spherical aerodynamic equivalent diameter with the same physical properties at which half the mass lies below the stated diameter.

**Results**

Particle sizes were determined for the nebulized isotonic and hypertonic saline solutions carried by different air conditions and flow rates and are presented in Supplemental Figure 1. The effect of condensation growth is apparent in the larger particle size created when the carrier air was 38 °C with dew point 38 °C (100% relative humidity), also called “body temperature pressure saturated” (BTPS) air.

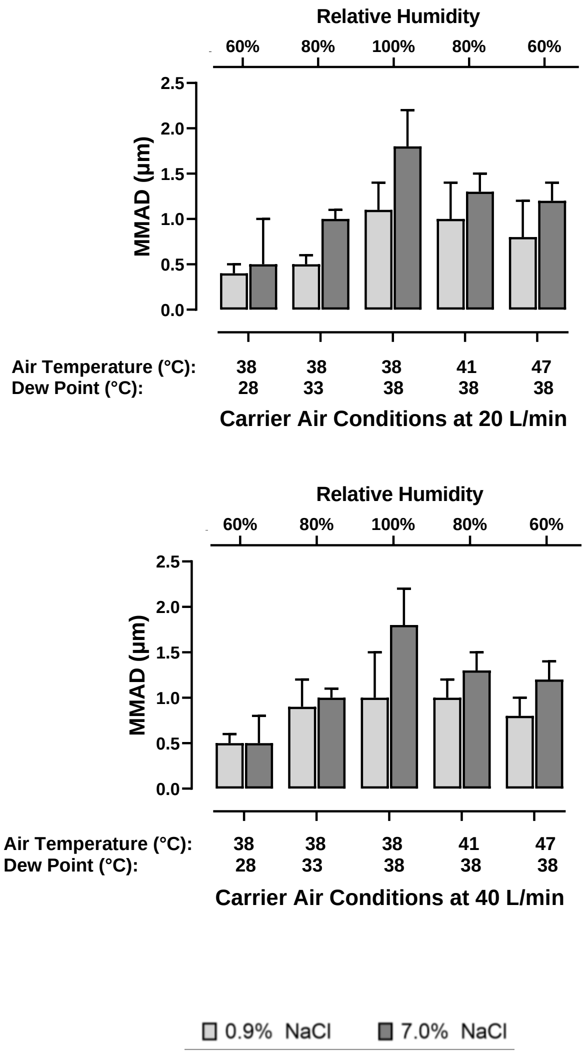


**Supplemental Figure 1:** Effect of nebulized 0.9% NaCl (light grey) and 7.0% NaCl (dark grey) on the mass median particle diameter (MADD) under different carrier air conditions at 20 L/min (top) and 40 L/min (bottom) delivered by a nasal high-flow system. MMADs from NaCl concentrations were significantly different across the carrier air conditions and flow rates.

A two-way ANOVA Tukey test was performed to analyse the effect of flow rate and carrier air conditions on the MMAD with nebulized 0.9% NaCl and 7.0% NaCl. This revealed that there was not a statistically significant interaction between the effects of the flow rate and carrier air condition ($F (4, 20) = 0.65$, $p = 0.64$ for 0.9% NaCl and $F (4, 20) = 1.091$, $p = 0.39$ for 7.0% NaCl).

Simple main effects analysis showed that the flow rate did not have a statistically significant effect on the MMAD for either 0.9% NaCl or 7.0% NaCl ($p = 0.46$, and $p = 0.67$, respectively). However, such analysis revealed that the carrier air conditions did have a statistically significant effect on the MMAD with 0.9% NaCl and 7.0% NaCl ($p = 0.01$, and $p < 0.0001$, respectively).

**Supplemental Table 2:** Two-way ANOVA analysis of mass median particle diameter (MMAD) changes with NaCl concentration, flow rate and carrier air conditions.

| [NaCl] | ANOVA table | SS | DF | MS | F (DFn, DFd) | P value |
|---|---|---|---|---|---|---|
| 0.9% | Interaction | 0.2220 | 4 | 0.05550 | F (4, 20) = 0.6453 | 0.6366 |
| | Air conditions | 1.410 | 4 | 0.3525 | F (4, 20) = 4.099 | 0.0138 |
| | Flow rate | 0.04800 | 1 | 0.04800 | F (1, 20) = 0.5581 | 0.4637 |
| | Residual | 1.720 | 20 | 0.08600 | - | - |
| 7.0% | Interaction | 0.2880 | 4 | 0.07200 | F (4, 20) = 1.091 | 0.3878 |
| | Air conditions | 3.468 | 4 | 0.8670 | F (4, 20) = 13.14 | < 0.0001 |
| | Flow rate | 0.01200 | 1 | 0.01200 | F (1, 20) = 0.1818 | 0.6744 |
| | Residual | 1.320 | 20 | 0.06600 | - | - |

**Volume change from particle deposition or osmolarity calculations**

Using the particle size generated by each NaCl solution under BTPS conditions, the volume change on the ASL, determined from the ASL level measured by the displacement sensor, can be approximated as either being solely from the particle deposition or a combination of deposition and osmotic action.

Iso-osmolarity has been established as 330 mOsm[7] with a mean ASL level of 21 µm, made up of the periciliary layer (approximately 7 µm) and mucus layer[8].

Exposure to isotonic and hypertonic saline resulted in an ASL-height change. As shown below, the authors determined how much particle deposition is required under each condition to result in the measure change to the ASL height. Using the particle size generated

at 20 L/min with carrier air at BTPS conditions, the following illustrations show the change in volume (blue) over a 1 μm$^2$ surface area.

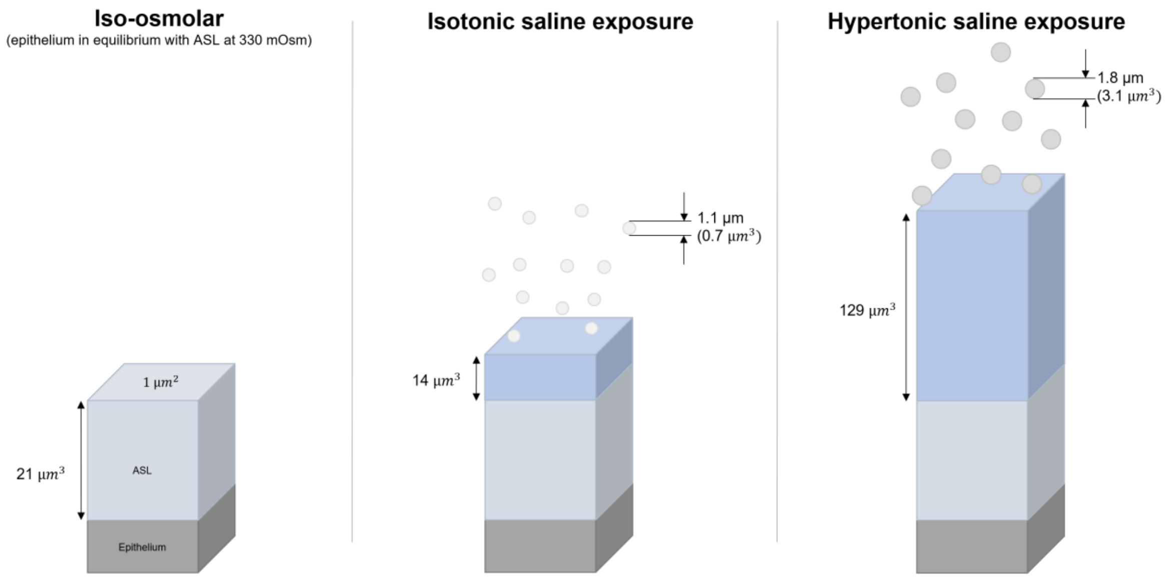


To determine the number of particles generated during nebulization, the authors assumed no losses, where the volume of NaCl solution placed in the Aerogen nebulizer (7.5 mL) over a 15-minute period was the volume nebulized into the particle size measured above (Supplemental Figure 1) for each NaCl concentration (0.9 and 7.0%) at 20 L/min with BTPS carrier air.

$$\frac{7.5\ mL}{15\ min} = 0.5 mL/min = 5x10^{11}\ \mu m^3/min$$

Accordingly, the particles generated from the isotonic (0.9% NaCl) and hypertonic (7.0% NaCl) solutions over an assumed tracheal surface area of 30 cm long and 10 cm wide for 15 minutes are calculated below, respectively:

$$\frac{5 \times 10^{11}\ \mu m^3}{min}\frac{particle}{0.7\ \mu m^3}\frac{1}{3\ \times 10^{10}\ \mu m^2}\frac{15\ min}{1} = 356\ particles/\mu m^2$$

$$\frac{5 \times 10^{11}\ \mu m^3}{min}\frac{particle}{3.1\ \mu m^3}\frac{1}{3\ \times 10^{10}\ \mu m^2}\frac{15\ min}{1} = 81\ particles/\mu m^2$$

The number of particles that would need to be deposited on the 1 $\mu m^2$ surface area to achieve the measured change in ASL level when the epithelium was exposed to isotonic (0.9% NaCl) nebulized saline is calculated as:

$$\frac{particle}{0.7\ \mu m^3}\frac{14\ \ \mu m^3}{\Delta\ ASL\ level} = 20\ particles\ deposited\ to\ change\ the\ ASL\ level$$

When the epithelium was exposed to isotonic saline, 6% (20/356) of the particles generated would need to be deposited over a 1 $\mu m^2$ surface area to achieve the change in ASL height measured. This value is approximately in line with values reported in Ari et al.[9], where the amount of particles emitted by the Aerogen nebulizer when placed before the humidifier in a similar experimental set-up was 7%.

With hypertonic saline (7.0% NaCl), the number of particles that would need to be deposited over a 1 $\mu m^2$ surface area to achieve the measured change in ASL level is calculated as:

$$\frac{particle}{3.1\ \mu m^3}\frac{129\ \ \mu m^3}{\Delta\ ASL - thickness} = 42\ particles\ deposited\ to\ change\ the\ ASL\ level$$

As a portion of the total particles generated, 52% (41/82) of particles would need to be deposited to achieve the change in ASL height measured during exposure to hypertonic (7.0%) nebulized saline. This number is much greater than those seen in the literature, and, therefore, it appears that the large change in ASL level is a combination of a volume change caused by particle deposition and osmotic action, drawing water into the ASL from the underlying epithelium.

Further calculations were performed to determine the change in ASL level attributed to particle deposition, assuming 7% of particles can be deposited onto the ASL surface to cause a change in the ASL height, and the ASL-level change resulting from osmotic action.

Assuming a 7% particle deposition, the change in ASL level would be:

$$\frac{7}{100}\ \frac{81\,particle}{\mu m^2}\ \frac{3.1\ \mu m^3}{particle} = 18\ \mu m$$

If 18 µm of the ASL-height change is attributed to particle deposition, 111 µm of the ASL-level change is from osmotic action.

Assuming the particles generated by the hypertonic (7.0% NaCl) solution have the same concentration, and the ASL remains iso-osmolar, the number of particles deposited into the 111 µm ASL-height change is:

$$\frac{70\,mg\ NaCl}{1000\,mL}\ \frac{1\,mL}{1\ \times 10^{12}\ \ \mu m^3}\frac{3.1\ \mu m^3}{particle} = 2.2\ \times\ 10^{13}\ mg\ NaCl/particle$$

$$\frac{70\,mg\ NaCl}{1000\,mL}\ \frac{1\,mL}{1\ \times 10^{12}\ \ \mu m^3}\frac{111\ \mu m^3}{ASL\ level} = 7.8\ \times\ 10^{12}\ mg\frac{NaCl}{ASL} - thickness$$

$$\frac{7.8\ \times\ 10^{12}\ mg\ NaCl/ASL\ level}{2.2\ \times\ 10^{13}\ mg\ NaCl/particle} = 35\,particle$$

Therefore, 35 particles of 7.0% NaCl concentration need to be deposited onto the ASL surface to cause an osmotic change on the ASL height, increasing it by 111 µm. This is still 43% of the total number of particles emitted, much greater than the amount of 7% shown in past literature. As such, osmotic action appears to play a part in drawing water into the ASL to achieve the change in ASL height recorded during the study.

## Conclusions

The carrier air condition had an effect on the particle size while the air-flow rate did not have a significant effect on the particle size. The saline concentration and relative humidity had a significant effect on particle sizes in all cases, except when nebulized isotonic saline was exposed to warmer air (heated above 37 °C) with 80% and 60% relative humidity (dew point 37 °C). Condensation growth of the particles, when carried by BTPS air, significantly reduced their salinity, resulting in very minor increases to the osmolarity of the ASL.

## Part 4: Mathematical modelling to estimate the thermal effects from condensation on the surface and changes to the airway surface liquid (ASL) film height

The airway surface is known to be sensitive to changes in temperature. By measuring the surface temperature using infrared-imaging, the authors saw a drop in surface temperature when the airway surface was exposed to the nebulized saline solutions. A larger decrease in temperature was seen when nebulized saline was entrained into body temperature and pressure saturated (BTPS) air at high flow (20 L/min).

Presented below is a mathematical model to enable a better understanding of the thermal effects that result from particles condensing onto the airway surface, and how the changes in the airway surface film height play a part in the heat transfer.

When heated and humidified air comes into contact with a surface that has a lower temperature than the dew point temperature of the air, it condenses into a liquid releasing latent heat. The heat transfer accompanied by condensation is termed “condensation heat-transfer”. When considering what happens to the water vapour in the air coming into contact with the airway surface, the term “film-wise condensation” describes the heat transfer taking place. In film-wise condensation, a saturated single-component (water) vapour condenses and

forms a continuous film on the airway surface where the heat transfer is controlled by conduction in the liquid film.

**Heat-transfer estimate equations**

When condensation occurs on the airway surface, the film height on the surface is thin and so the curvature of the cylindrical airway in the trachea can be disregarded. A schematic diagram of the film-wise condensation on the airway surface is shown below (Figure 1) with the parameters in the following calculations used to estimate the heat transfer at the gas-liquid interface when air of different conditions comes into contact with the airway surface.

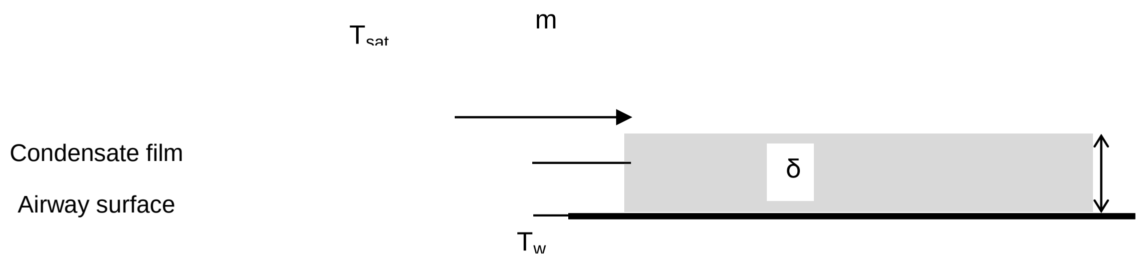


where $T_w$ is the wall temperature (°C), $T_{sat}$ is the saturation temperature of the air (°C), δ is the film height (mm) and m is the air-flow rate (L/min).

Assuming friction forces are negligible on the surface and the movement along the airway surface by mucus transport velocity (MTV) is negligible compared to the air-flow rate (m), the boundary-layer conditions can be approximated as:

Continuity:

$$\frac{\partial u}{\partial x} + \frac{\partial v}{\partial y} = 0 \qquad \text{(Equation 1)}$$

Conservation of momentum:

$$\rho_l u \frac{\partial u}{\partial x} + \rho_l v \frac{\partial u}{\partial y} = \mu_l \frac{\partial^2 u}{\partial y^2} + (\rho_l - \rho_g) m \qquad \text{(Equation 2)}$$

Conservation of energy:

$$c_{pl} \rho_l u \frac{\partial T}{\partial x} + c_{pl} \rho_l v \frac{\partial T}{\partial y} = k_l \frac{\partial^2 T}{\partial y^2} \qquad \text{(Equation 3)}$$

where $\rho_l$ is the density of water, $\rho_g$ is the density of vapour, $\mu_l$ is the viscosity coefficient at of water, $c_{pl}$ is the specific heat at constant pressure of the water, $k_l$ is the thermal conductivity of water and $m$ represents the mass flow rate of the air.

Using the schematic diagram shown above, the boundary conditions are as follows:

$u = v = 0,\ T = T_w$ at $y = 0$ (the airway surface)

$\mu_l \frac{\partial u}{\partial y} = 0, T = T_{sat}$ at $y = \delta$ (the condensate film surface)

As the film is considerably thin and the velocity of the film (MTV) is small, the transport momentum due to convection can be disregarded. Therefore, the left side of the conservation of momentum equation (Equation 2) can be approximated to zero, resulting in:

$$\mu_l \frac{\partial^2 u}{\partial y^2} = -(\rho_l - \rho_g) m$$

By integrating the above equation and determining the integration constants from the boundary conditions, the velocity distribution in the liquid film can be described as:

$$u = \frac{(\rho_l - \rho_g) m}{2\mu_l} (2\delta - y^2)$$

Because the transport momentum can be approximated as zero, similarly the energy transport due to convection can be estimated as being zero, which results in the conservation of energy equation (Equation 3) becoming:

$$\frac{\partial^2 T}{\partial y^2} = 0$$

By integrating the above equation, the temperature distribution in the film can be defined as:

$$T = T_w - (T_w - T_{sat})\frac{y}{\delta}$$

When considering the energy balance of the condensing vapour over the liquid film flowing from position $x = 0$ to $x = x$, using the latent heat of vapourization $h_{fg}$, the total amount of latent heat released during condensation relative to the total heat transferred through the airway surface can be determined as:

$$h_{fg}\rho_l \int_0^{\delta} u dy = \int_0^{x} k_l \left(\frac{\partial T}{\partial y}\right)_{y=0} dx$$

The local heat-transfer coefficient at position $x$ ($h_x$) can be determined using the equation that defines the temperature distribution in the film ($T = T_w - (T_w - T_{sat})\frac{y}{\delta}$) and the energy balance of the condensing vapour over the liquid film ($h_{fg}\rho_l \int_0^{\delta} u dy = \int_0^{x} k_l \left(\frac{\partial T}{\partial y}\right)_{y=0} dx$) to give:

$$h_x = \frac{-k_l \left(\frac{\partial T}{\partial y}\right)_{y=0}}{(T_w - T_{sat})} = \frac{k_l}{\delta}$$

From the above equation, it is evident that the heat transfer is controlled by thermal conductivity ($k_l$) (heat conduction) and height of the film ($\delta$).

## Calculations

The following table shows the heat-transfer ($h_x$) estimates determined from the measurements used in the study, where the surface temperature of the film ($T_{sat}$) was used to adjust the thermal conductivity of the liquid film ($k_l$) under each condition and the measured relative change in film-height level was used for the film-height measurement ($\delta$).

| Air condition | $T_{sat}$ (°C) | $k_l$ (W/mK) | δ (mm) | $h_x$ (W/mm) |
|---|---|---|---|---|
| High-flow nebulized isotonic saline | 37.7 | 0.6253 | 0.0368 | 17.0 |

| | | | | |
|---|---|---|---|---|
| High-flow nebulized hypertonic saline | 36.4 | 0.6235 | 0.1672 | 3.7 |
| Low-flow nebulized isotonic saline | 37.8 | 0.6255 | 0.0578 | 10.8 |
| Low-flow hypertonic saline | 36.4 | 0.6235 | 0.1592 | 3.9 |

**Conclusions**

In general, the study showed that thinner airway surface films had larger heat transfers than thicker airway surface films. The airway surface film height was found to be more dependent on the NaCl concentration than flow rate where isotonic saline resulted in thinner airway surface films compared with exposure to hypertonic saline.

The change in surface temperature had little effect on the calculated heat transfer where the largest heat transfer reported (17 W/mm) resulted from a 0.9 °C surface temperature decrease when exposed to nebulized isotonic saline with high-flow carrier air. However, the largest surface temperature drop (2.2 °C) was recorded when nebulized hypertonic saline was used to deliver with high-flow air, resulting in just 3.7 W/mm heat transfer. The larger changes in surface temperature appeared to be more dependent on the flow rate where high flow caused more evaporative cooling than those recorded with low flow.

Flow rate and the height of the airway surface film appeared to have the largest effect on the calculated heat transfer where the airway surface film height was most affected by the osmotic gradient, which was larger when nebulized hypertonic saline was introduced. While the heat-transfer effects seemed to be fewer than when hypertonic saline was used, these thermal effects may have been overshadowed by the larger osmotic action seen by the change in ASL level in Figure 3 of the main text section.

## Part 5: Effect of cooler and drier air on the ASL and mucociliary transport

To determine the effects of air colder and drier than body temperature pressure saturated (BTPS) air on the ASL height and mucociliary transport, the authors used infrared video microscopy and a displacement sensor to study sheep tracheas exposed to air at BTPS followed by air that was colder and drier (31 °C) with 60% relative humidity for 15 minutes. An understanding of the effects of temperature and humidity on the ASL and mucociliary transport is essential to describe the functioning of the tracheal mucosa in intubated patients.

**Methods**

Five tracheas, harvested from apparently healthy sheep immediately after slaughter, were collected from an abattoir and transported at room temperature to the laboratory. The tracheas were opened along the ventral mid-line and fixed flat with the epithelium positioned upward in a previously described[1] heated (38 °C) organ bath (Figure 1). Time from collection at the abattoir to the beginning of the laboratory experiments was around 30 minutes for each trachea.

An air-flow generator with humidifier (Airvo 2, Fisher & Paykel Healthcare, New Zealand) was used to create a unidirectional flow of heated and humidified air over the epithelial surface of the tracheas at 20 L/min (high-flow delivery) to mimic nasal high-flow delivery. The temperature and dew point of the air were controlled using external sensors at the inlet of the organ bath (HMP110, Vaisala, Finland).

The top cover of the organ baths contained heated $CaF_2$ optical windows, one of which was used to continuously measure the ASL with a displacement laser sensor (LK-G32 and LK-Navigator software, Keyence Corporation, Japan). This sensor is based on the light-scattering principle and has repeatability of up to 0.05 µm. Its analogue signal was recorded using Keyence software (LK-Navigator, Japan). The other optical window was used for simultaneous IR high-speed macro-video imaging (SC7000 camera and L0905 lens, FLIR,

US) to measure MTV, CBF and surface temperature, as reported in detail previously[2]. The IR macro-video imaging was carried out for 30 seconds, every 5 minutes, during the 30-minute experiment, at 100 frames/s with a field of view of 9.6 x 7.7 mm.

Baseline measurements were made during the first 15 minutes with air heated to 38 °C and fully saturated with water (BTPS) on all tracheas (N = 10). Thereafter measurements were made at 15-minute intervals with exposure to reduced temperature and dew-point air conditioned to 31 °C and 60% relative humidity (23 °C dew point).

GraphPad Prism (V8.3.0, US) was used for statistical analysis of measurements. Significance testing was performed using a two-tailed paired t-test, and two-way ANOVA Tukey test where $p < 0.05$ was statistically significant. All data was tested for normality with the D'Agostino & Pearson test.

**Results**

During the baseline period (t = 0 to 15 minutes), the mean surface temperature (37.9 ± 0.1 °C (Figure 2), MTV (8.2 + 0.8 mm/min) and CBF (13.1 + 0.6 Hz) (Figures 1 and 2) were within normal ranges previously described[1]. Air with reduced temperature (31 °C) and humidity (dew point 23 °C) at the high-flow rate (20 L/min) significantly ($p < 0.05$) lowered the airway surface temperature (31.4 + 0.5 °C), MTV (4.6 + 4.1 mm/min) and CBF (8.1 + 0.7 Hz) after 15 minutes. The drop in surface temperature and mucociliary transport was accompanied by a substantial reduction in the change in ASL thickness (-123.6 ± 46.3 µm).

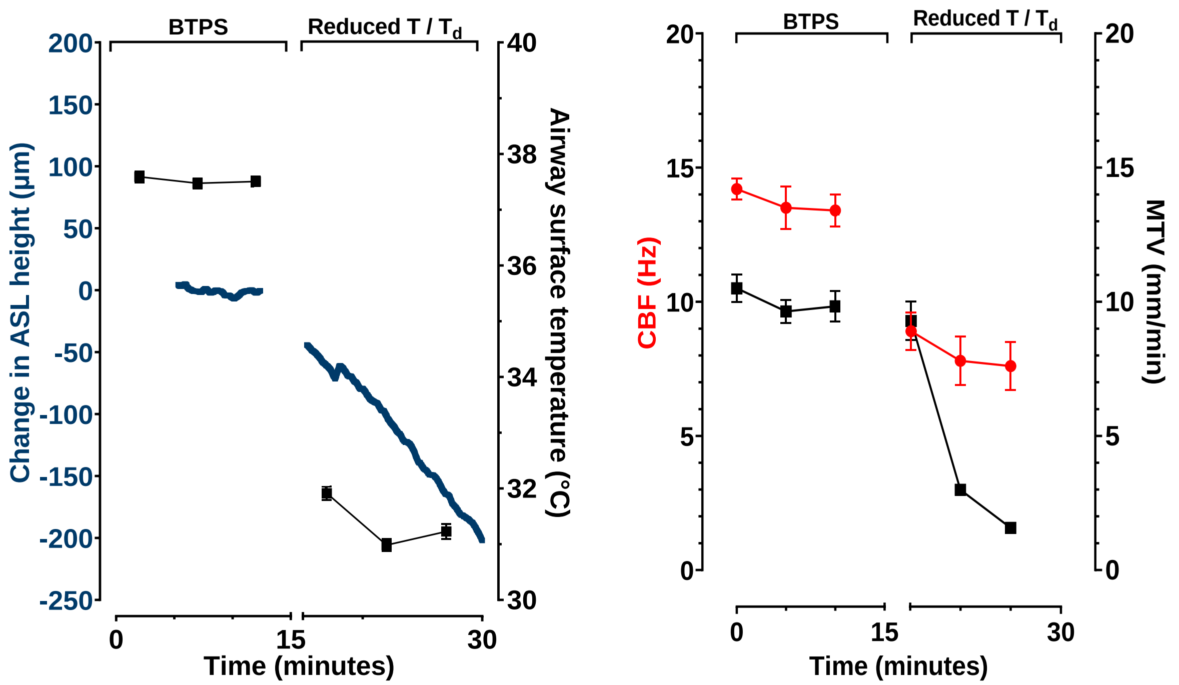


**Supplemental Figure 2.** Representative real-time airway surface liquid (ASL) heights (blue) and 5-minute mean airway surface temperatures (black), cilia beat frequency (CBF) (red) and mucus transport velocity (MTV) (black) measured over a 30-minute period on the airway surface exposed to body temperature and pressure saturated (BTPS) air (38 °C, dew point 38 °C) for the first 15 minutes, followed by 15 minutes of exposure to reduced temperature (T) and dew point ($T_d$) air conditioned to 31 °C and 60% relative humidity (23 °C dew point).

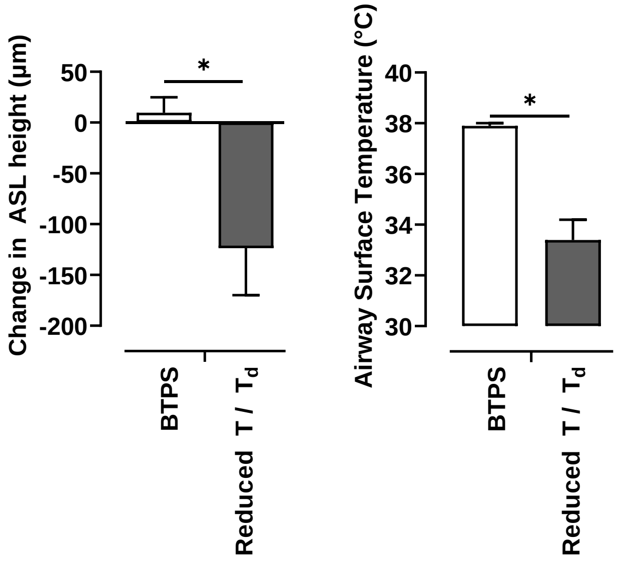

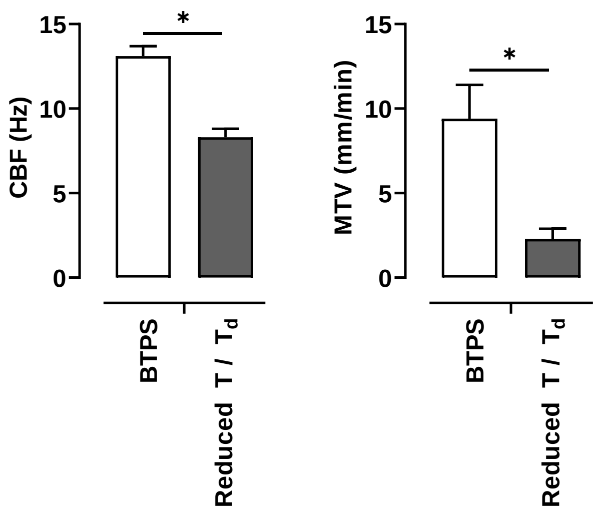


**Supplemental Figure 3.** Mean effect of reduced temperature (T) and dew point ($T_d$) air conditioned to 31 °C and 60% relative humidity (23 °C dew point) compared to body temperature and pressure saturated (BTPS) air (38 °C, dew point 38 °C) baseline conditions (white) on the change in the airway surface liquid (ASL) height, airway surface temperature, cilia beat frequency (CBF) and mucus transport velocity (MTV) on the airway surface (5 tracheas). Airway surface temperatures, CBF and MTV were measured with infrared video-imaging while simultaneous measurements of the ASL were made with a displacement laser sensor. Mean ASL heights presented above are from the last 5 minutes of the experiment. * = significant difference.

## Discussion

The response to low temperature and humidity air was consistent with previous reports showing impaired mucociliary transport[1] and a desiccated ASL[10]. Of note is the magnitude of the change in the ASL thickness, which decreased by approximately 100 µm, suggesting the change in the ASL thickness in response to cooler and drier air causes not only the ASL to be dehydrated but also the epithelial cells below to become desiccated.

## REFERENCES


1. Kelly, S.J., P. Martinsen, and S. Tatkov, *Rapid changes in mucociliary transport in the tracheal epithelium caused by unconditioned room air or nebulized hypertonic saline and mannitol are not determined by frequency of beating cilia.* Intensive Care Med. Exp., 2021. **9**(1): p. 8.
2. Kelly, S.J., et al., *Variability in tracheal mucociliary transport is not controlled by beating cilia in lambs in vivo during ventilation with humidified and non-humidified air.* Am. J. Physiol. - Lung Cell., 2021.
3. Longest, P.W., J.T. McLeskey, and M. Hindle, *Characterization of Nanoaerosol Size Change During Enhanced Condensational Growth.* Aerosol Sci. Technol., 2010. **44**(6): p. 473-483.
4. Darquenne, C., *Aerosol deposition in health and disease.* Journal of aerosol medicine and pulmonary drug delivery, 2012. **25**(3): p. 140-147.
5. Hicks, J.F., et al., *Measurements of growth due to condensation for some common aerosols.* J. Aerosol Sci., 1989. **20**(3): p. 289-292.
6. Hindle, M. and P.W. Longest, *Evaluation of enhanced condensational growth (ECG) for controlled respiratory drug delivery in a mouth-throat and upper tracheobronchial model.* Pharm. Res., 2010. **27**(9): p. 1800-11.
7. Jayaraman, S., Y. Song, and A.S. Verkman, *Airway surface liquid osmolality measured using fluorophore-encapsulated liposomes.* J Gen Physiol, 2001. **117**(5): p. 423-430.
8. Jayaraman, S., et al., *Noninvasive in vivo fluorescence measurement of airway-surface liquid depth, salt concentration, and pH.* J. Clin. Investig., 2001. **107**(3): p. 317-324.
9. Ari, A., et al., *Comparison of Aerosol Deposition with Heated and Unheated High Flow Nasal Cannula (HFNC) in Healthy Adults*, in *Am. Thorac. Soc.* p. A5320-A5320.
10. Alexopoulos, C., B. Jansson, and C.E. Lindholm, *Mucus Transport and Surface Damage After Endotracheal Intubation and Tracheostomy. An Experimental Study in Pigs.* Acta. Anaesthesiol. Scand., 1984. **28**(1): p. 68-76.
